# Cognitive impairment in schizophrenia across age groups: a case–control study

**DOI:** 10.1186/s12888-016-0749-1

**Published:** 2016-02-24

**Authors:** Anna Mosiołek, Jacek Gierus, Tytus Koweszko, Agata Szulc

**Affiliations:** Clinic of Psychiatry, Department of Health Sciences, Medical University of Warsaw, Ul. Partyzantów 2/4, Pruszków, 05-802 Poland

**Keywords:** Schizophrenia, Cognitive impairment, Executive functions

## Abstract

**Background:**

The potential dynamics of cognitive impairment in schizophrenia is discussed in the literature of the field. Recent publications suggest modest changes in level of cognitive impairment after first psychotic episode. Present article attempts to explore cognitive differences between patients and controls across age groups and differences between age groups in clinical group.

**Methods:**

One hundred and twenty-eight hospitalized patients with schizophrenia (64 women and 64 men) and 68 individuals from the control group (32 women and 32 men) aged 18–55 years were examined. The patients were divided into age groups (18–25, 26–35, 36–45, 46–55). Both groups were examined using Wisconsin Card Sorting Test, Rey Auditory Verbal Learning Test, Rey Osterrieth Complex Figure Test, Trail Making Test (A and B), Stroop Test, verbal fluency test and Wechsler digit span.

**Results:**

Patients with schizophrenia obtained significantly lower scores versus the control group in regard to all the measured cognitive functions (Mann–Whitney U; *p* < 0.05. Most deficits were present in all age groups, however, statistically important impairment in executive functions (WCST) were present only in “older” groups.

**Conclusions:**

Patients with schizophrenia obtained less favourable results than the control group in all age groups. Deficits regarding executive functions do not seem to be at a significant level among the youngest group, whereas they are more noticeable in the group of 46–55-year-olds. Executive functions are significantly lowered in the group aged 36–45 in comparison to the “younger” groups. The level of cognitive functions shows a mild exacerbation in connection with age, whereas cognitive rigidity proved to be related to the number of years spent without hospital treatment.

## Background

In his pioneering research, Bleuler already noticed that the essence of schizophrenia are not hallucinations or delusions but the so-called axial symptoms that significantly affect the global functioning of patients. In her works, Andreasen [1] presented a neo-Bleulerian model, describing schizophrenia as a neurodevelopmental disorder with the occurrence of primary cognitive deficits corresponding to "axial" symptoms [1–4]. The neurodevelopmental irregularities present in schizophrenia may arise from the incorrect formation of large-scale neural networks, for example at the axonal level, but in most cases, the dysfunction occurs at the level of sending and receiving information via synapses or dendrites [5]. A consequence of these irregularities is the phenomenon of cognitive dysmetria associated with impaired coordination between thinking and action, leading to the distortion of such cognitive processes as memory, attention focus, language, emotional intelligence and awareness [2, 6].

Currently two main theories are presented regarding the dynamics of the disease process in schizophrenia. The first theory assumes a gradual stabilization of the mental condition in a relatively short period of time (2–5 years) from the onset, or the transition to the stationary form of schizophrenia [7]. This hypothesis provides for the possibility of a slight improvement in the general psychopathology and cognitive impairments. The second theory considers a gradual build-up of impairment beginning in the prodromal phase where the cognitive dysfunctions, discrete negative symptoms and behaviour disorders are visible through the acute phase of the disease with the occurrence of severe positive symptoms. In this theory, the subsequent phases of the disease include the occurrence of periods of remission and exacerbation with progressively aggravated course, leading to the final phase of schizophrenia with predominantly negative symptoms and intensified cognitive impairment [8, 9]. This hypothesis would therefore assume the progression of cognitive deficits associated with time from the onset of the first symptoms and age.

The cognitive impairments present in schizophrenia most frequently cited in the literature include impairments of working memory, executive functions, verbal fluency, attention, and disturbances in the selection and processing of information [10–12]. It is believed that this type of disorders are present in 85 % of the patients and are negatively correlated with psychosocial functioning and cooperation in therapy [10]. The research most often emphasizes their specific and selective character in schizophrenia [13–15] and does not confirm the increase in cognitive deficits during the course of the disease, but rather their stability and independence from the clinical picture [7]. Numerous previous and contemporary studies proved the occurrence of cognitive disorders many years before the onset of the disease, frequently even in childhood [16–20]. The occurrence of cognitive disorders confirmed with neuropsychological tests is a more specific marker of early schizophrenia, than for example, diagnostics by means of structural MRI (sMRI) [21].

The occurrence of cognitive deficits in schizophrenia may be explained by the glutamate hypothesis. Cognitive disorders can be a consequence of the neurotoxic activity of the psychosis and the external factors [8, 9, 22] and some researchers believe that the progressive process of neuronal degeneration and the associated increase in "residual" symptoms may be the result of excessive glutamatergic transmission. According to them, in the context of excessive excitation of the glutamatergic transmission a toxic neurodegenerative excitation could occur, in which the NMDA receptor would be involved. In theory, as a result of the initiated pathological process this normal neuronal excitation process would lead to a random toxic excitation that would in turn lead to neuronal damage due to slow neurodegeneration resulting from the opening of the calcium channels, secondary activation of the intracellular enzymes, and generation of free radicals toxic to cell membranes and intracellular structures [8, 9, 23]. Consequently, this would lead to the overlapping of primary cognitive deficits with secondary cognitive disorders, associated with the phenomenon of excitotoxicity and neurodegeneration. The phenomenon of neurodegeneration is reflected in the neuroimaging studies of patients with long-term course of schizophrenia. In the group of patients with schizophrenia there is a decline in the volume of the gray matter and the white matter, which is associated with age exacerbations. In case of gray matter an overall reduction of its volume is observed with particular intensity in the frontal cortex and the thalamus area whereas white matter atrophic changes relate mainly to the frontal, temporal and parietal regions. The widening of lateral ventricles and the enlargement of fluid spaces in the frontal, temporal and parietal regions were also observed in longitudinal brain MRI studies [24]. The above-mentioned changes were correlated with cognitive impairment, but had little impact on other clinical parameters [16, 24–26].

At the same time, the results of some meta-analyses show a similar role being played by factors other than the course of the disease, such as the influence of medications or overuse of psychoactive substances. These meta-analyses suggest that there is no clear proof for the neurodegenerative nature of schizophrenia [27]. At the same time, a study combining longitudinal and cross-sectional methodologies shows a similar level of cognitive disorders in patients after their first schizophrenia episode to those with an established clinical picture of the disease [27]. Individual studies also show the presence of other factors, such as metabolic syndrome, lowered BDNF, improper diet, low level of physical activity or stigma [28]. The reversibility of processes causing lowered levels of functioning is also shown in research on rehabilitation, both in terms of symptoms and the social aspect, where patients experience tangible benefits when cognitive remediation training is included as a part of comprehensive psychiatric rehabilitation [29–31]. Complex rehabilitation proved to have an impact on global functioning and global cognitive improvement was a mediator for improvements in functional outcomes [30]. Many patients can then recover or benefit from proper rehabilitation.

Therefore, it is important to clarify whether the cognitive disorders in schizophrenia exacerbation are static or progressive with the duration of the disease and whether they are associated with the patient's age or other factors (duration of the disease, medication, number of hospitalizations, years of education, difference between years from onset and years form 1^st^ hospitalization). Variable intensity relationships can be expected between cognitive functions and aforementioned factors. This study pertains to a conducted correlation analysis between the cognitive tests results and aforementioned factors. It is expected that statistically significant differences occur between clinical and control groups, whereas the strength and number of those differences should grow along with age in the individual age groups. The main objective of the study is to analyse the differences in the number and type of cognitive functions which are lowered in case of people with schizophrenia with regard to the control group for individual age groups. It can be expected that the lowering of cognitive functions will be visible in all age groups, although it is difficult to predict in which areas it will be particularly noticeable.

The present study aimed to determine the seriousness of the dysfunctions suffered by patients with schizophrenia, whether these “deficits” [3] (in comparison to controls) are global vs. selective, or whether they are “more visible” across age groups.

## Methods

### Examined groups

The study was conducted one time in a group of 128 patients (64 W + 64 M) aged 18–55 years, hospitalized with a diagnosis of paranoid schizophrenia. The numbers of patients in individual age groups are presented in Table 1.

**Table 1:** The number of subjects in individual age groups (clinical and control groups)

Age group	Patients with schizophrenia (*N* = 128)	Healthy controls (*N* = 68)
18–25	32	22
26–35	32	21
36–45	32	12
46–55	32	13

The diagnosis was made on the basis of DSM-IV-TR criteria [27]. The control group consisted of 64 healthy individuals (32 W + 32 M) matched for sex and age with the clinical group. A minimum education threshold of 12 years was determined for both groups. Patients with psychiatric co-morbidity, sight or hearing disorders, movement disorders or chronic somatic illness were excluded from the study. All patients participating in the study met the criteria for psychotic exacerbation measured using the PANSS scale [30].

In the studied group of 128 patients with schizophrenia the number of hospitalizations in the individual groups (18–25, 26–35, 36–45, 46–55) increased with the age of patients and the duration of the disease process. The length of the current hospitalization did not differ in a statistically significant manner in the individual age groups and the mean was 6.5 weeks. The age and sex of the examined patients was controlled. The mean time that elapsed since the first hospitalization was 1.25 years in the youngest age group and reached 14.31 years in the oldest group (the mean for the entire group was 7.7 years). The mean time that elapsed from the onset of these symptoms to the first hospitalization was determined on the basis of a thorough history taking oriented towards the occurrence of increasing prodromal disorders. In patients diagnosed with schizophrenia, who participated in the study it was 1.4 years. More clinical data is illustrated in Table 2.

**Table 2:** Selected clinical and demographic data—patients with schizophrenia

Age group			
18–25	Education (years)	*M* = 12,546	sd = 1,700
	Children	*M* = 0,000	sd = 0,000
	Number of hospitalizations	*M* = 2,187	sd = 1,635
	Years from 1st hospitalization	*M* = 1,250	sd = 1,736
	Years from onset of disease	*M* = 2,406	sd = 2,284
26–35	Education (years)	*M* = 13,406	sd = 2,241
	Children	*M* = 0,218	sd = 0,6082
	Number of hospitalizations	*M* = 5,187	sd = 4,828
	Years from 1st hospitalization	*M* = 4,156	sd = 4,141
	Years from onset of disease	*M* = 5,046	sd = 4,028
36–45	Education (years)	*M* = 12,312	sd = 2,292
	Children	*M* = 0,750	sd = 1,218
	Number of hospitalizations	*M* = 10,312	sd = 11,487
	Years from 1st hospitalization	*M* = 10,968	sd = 7,310
	Years from onset of disease	*M* = 12,562	sd = 7,052
46–55	Education (years)	*M* = 12,375	sd = 2,075
	Children	*M* = 1,187	sd = 1,856
	Number of hospitalizations	*M* = 10,000	sd = 11,210
	Years from 1st hospitalization	*M* = 14,312	sd = 7,997
	Years from onset of disease	*M* = 16,645	sd = 7,918

Statistically significant differences between patients and healthy individuals were revealed regarding: the level of education, professional activity, remaining in stable relationships and having children. All patients participating in the study met the criteria for psychotic exacerbation. The mean total PANSS score was 78.4 (SD = 17.6) points. The distribution of the mean scores in individual age groups was as follows: 1) 84.34; 2) 75.28; 3) 73,84; 4) 80.06. The correlations of the PANSS results and the neurocognitive tests were slight or weak in the whole group of patients (*N* = 128). Only in the case of the G7 scale (PANSS)—motor retardation and the verbal part of the Stroop test a moderately high negative correlation occurred (−0.58). The relationship between the clinical picture and neurocognitive variables was not confirmed in the study. Patients in the examined age groups were treated with both atypical and classical neuroleptics in oral and depot forms. For statistical purposes, all doses of medications administered to patients were converted to chlorpromazine equivalents in accordance with the applicable standards. The mean dose of the applied treatment expressed as chlorpromazine equivalent was 660 mg and in the individual age groups (in ascending order): 1) 493.04 mg; 2) 579.12 mg; 3) 446,38 mg; 4) 528,96 mg;. The maximum administered dose was 1633 mg and the minimum was 75 mg expressed as chlorpromazine equivalent. Sixty-two percent of the examined patients received second-generation medications and the most commonly administered neuroleptics were: olanzapine, clozapine, risperidone, and perphenazine. The mean total score of the MADRS scale for the whole group of patients was 11.9 (SD = 8.9). The patients obtained the highest score in the MADRS [32] scale in two categories: subjective assessment of depression and difficulties in focusing attention.

### Applied methods

A demographic—descriptive questionnaire, PANSS scale [30], MADRS scale, Wisconsin Card Sorting Test [33], Rey Auditory Verbal Learning Test, Rey-Osterrieth Complex Figure Test (ROCFT), TMT A [34], TMT B, Wechsler digit span and a verbal fluency test were used in the study.

PANSS (Positive and Negative Syndrome Scale) is a method created 1987 to measure the level of symptoms of schizophrenia, commonly used in studies on pharmacotherapy. MADRS (Montgomery-Asberg Depression Rating Scale) is a scale for evaluation of the level of depression, filled in by a clinician. Wisconsin Card Sorting Test (WCST) is a neuropsychological test used to measure executive functions, particularly mental flexibility in light of changing stimuli. Rey Auditory Verbal Learning Test (RAVLT) is used to measure the verbal and lingual ability to learn (acquiring, storing and retrieving verbal material). Rey-Osterrieth Complex Figure Test (ROCFT) is a method used to measure visual and cognitive functions, as well as perceptual organisation. Trail Making Test versions A and B (TMT A and TMT B) are tests used to measure visual attention and task switching. Wechsler Digit Span is a part of Wechsler's battery used to measure rote memory, whereas the test of fluency in terms of vocabulary measures the ability to retrieve words from memory and update them.

The RAVLT test examination procedure consisted in the examiner reading a series of 15 words (List A), from which the participant was supposed to recall as many words as possible. This procedure was repeated five times to give the examined individual the opportunity of learning the examination material. Subsequently a different list (list B) was read once with the instruction to remember as many words as possible. Later, the examined individual was asked to recall the words from list A from memory. After a 30 min interval, the examined individual was asked to recall without prompting the words from A list. The number of individual words in the series A1-A5, in the list B, delayed recalls with and without time interval were taken as the test performance metrics. In the ROCFT test the examined individuals were asked to copy a complex figure and then reproduce it from memory after 3–5 min. The number of correctly reproduced elements of the figure was adopted as the performance metric. In TMT A and B, the time of the correct performance of each part of the test was adopted as the performance metric. In the test of verbal fluency, the number of words recalled in 1 min was adopted as the performance metric. In Wisconsin Card Sorting Test the 64-card version was used, in which the examined individual had to match cards according to one of three possible criteria: color, geometric shape or number. In the WCST study the number of correct answers, sum of all errors committed, perseverative responses, perseverative errors, other errors, conceptual responses, number of attempts in the first category, sum of completed categories and the failure to maintain set were assessed. The number of items read within 1 min was taken as the performance metric of the Stroop test. The three typical parts of the test were used and the coefficient of interference was also calculated.

The Bioethics Committee of the Medical University of Warsaw was informed about the methodology of the study, procedure of consent, and approved its conduction. Patients have signed a consent to use their anonymised figures obtained through diagnostic tests in statistics required for this study. Anna Mosiołek deemed patients able to consent, basing on legal criteria of informed consent.

### Statistics

The Mann–Whitney *U* test was used to determine the significance of inter-group differences. Spearman's rank correlation coefficient was used to measure the correlations obtained in the study. Statsoft STATISTICA and SPSS Statistics 18 software was used for statistical analyses. In order to determine the level of significance of differences the Mann–Whitney *U* nonparametric test was used, whereas the determination of relationships’ strength between variables connected with the course of the disease and the level of cognitive functions was calculated using the Spearman's rank correlation coefficient.

## Results

### The relationships between the cognitive functions and the demographic variables and variables associated with the treatment process

A weak negative correlation between the years of education and the dose of administered medications (Rho = -0.177; *p* = 0.05) expressed as chlorpromazine equivalent seems interesting, whereas there is no correlation between the doses of medication and age.

Correlations presented in Tables 3-6 reflect mainly negative associations between executive functions and aging, and time from disease onset. Most of these correlations are weak. Weak relationships between the performance of the WCST and difference between years from onset and years from 1^st^ hospitalization were also found. Only rows and columns that contain significant or interesting correlations are presented in the tables.

**Table 3:** Relationship between the variables associated with the course of disease and the test results—correlations (Spearman’s rank correlation coefficient)—patients with schizophrenia

	WCST correct answers	WCST total errors	WCST perseverative responses	WCST perseverative errors	WCST conceptual level	WCST categories achieved	WCST trials to complete 1^st^ category	WCST failure to maintain set
Age	-,273^a^	,292^a^	,129	,081	-,256^a^	-,280^a^	,247^a^	-,213^b^
Education (years)	,051	-,051	-,050	-,051	,110	,001	,016	,219^b^
Hospitalizations	-,080	,094	,028	,010	-,104	-,189^b^	,142	-,102
Time from 1^st^ hospitalization	-,182^b^	,211^b^	,055	,043	-,202^b^	-,225^b^	,199^b^	-,182^b^
Time from onset of schizophrenia	-,203^b^	,213^b^	,085	,070	-,211^b^	-,218^b^	,224^b^	-,190^b^
Diffrerence between years from onset and years from 1^st^ hospitalization	-,069	,061	,185^b^	,200^b^	-,055	,083	-,050	-,082

^a^Correlation is significant at the 0.01 level (2-tailed)

^b^Correlation is significant at the 0.05 level (2-tailed)

**Table 4:** Relationship between the variables associated with the course of disease and the test results—correlations (Spearman's rank correlation coefficient)—patients with schizophrenia

	Wechsler forward	Wechsler backwards	Fluency A	Time Stroop words	Time Stroop colors	Time Stroop interferention	Stroop coefficient
Age	-,149	-,182^b^	-,032	-,254^a^	-,195^b^	-,283^a^	-,241^a^
Hospitalizations	-,101	-,106	-,040	-,163	-,196^b^	-,187^b^	-,206^b^
Time from 1^st^ hospitalization	-,178^b^	-,134	-,077	-,180^b^	-,262^a^	-,240^a^	-,244^a^
Time from onset of schizophrenia	-,193^b^	-,116	-,039	-,211^b^	-,218^b^	-,247^a^	-,246^a^

^a^Correlation is significant at the 0.01 level (2-tailed)

^b^Correlation is significant at the 0.05 level (2-tailed)

**Table 5:** Relationship between the variables associated with the course of disease and the test results—correlations (Spearman’s rank correlation coefficient)—patients with schizophrenia

	RAVLT A1-A5	RAVLT A6	RAVLT A7	RAVLT B	RAVLT Recognition
Age	-,270^a^	-,273^a^	-,227^b^	-,206^b^	-,332^a^
Children	-,086	-,046	-,030	-,185^b^	-,070
Hospitalizations	-,182^b^	-,215^b^	-,123	-,161	-,091
Time from 1^st^ hospitalization	-,308^a^	-,332^a^	-,243^a^	-,198^b^	-,229^b^
Time from onset of schizophrenia	-,310^a^	-,331^a^	-,253^a^	-,195^b^	-,298^a^

^a^Correlation is significant at the 0.01 level (2-tailed)

^b^Correlation is significant at the 0.05 level (2-tailed)

**Table 6:** Relationship between the variables associated with the course of disease and the test results—correlations (Spearman’s rank correlation coefficient)—patients with schizophrenia

	ROCFT similarity	ROCFT main elements	Time TMT A	Time TMT B
Age	-,218^b^	-,194^b^	,391^a^	,350^a^
Education (years)	,029	,080	-,195^b^	-,180^b^
Children	-,038	-,005	,206^b^	,080
Hospitalizations	-,121	-,099	,201^b^	,177^b^
Time from 1^st^ hospitalization	-,200^b^	-,176^b^	,326^a^	,303^a^
Time from onset of schizophrenia	-,197^b^	-,183^b^	,342^a^	,308^a^

^a^Correlation is significant at the 0.01 level (2-tailed)

^b^Correlation is significant at the 0.05 level (2-tailed)

Similar relationships are found in the case of digit span forward and digit span backwards tests, phonemic fluency, Stroop test, RAVLT, ROCFT and TMT. Very similar significant correlations (Spearman’s Rho values 0.24–0.31; *p* = 0.05) were detected between RAVLT test score and age, and Time of Stroop interferention subtest and age. Interestingly, the presented data didn’t reveal connection between score in WCST and age or education in the control group.

### Cognitive functioning—intergroup comparisons

The analysis of the obtained data began with a comparison of the clinical group versus the control group in regards to all measured cognitive functions, as presented in Table 7. Due to the unfulfilled requirements for the *t* test, the nonparametric Mann–Whitney *U* test was used. It was found that patients with schizophrenia obtained less favourable score in all possible attempts in all the tests. The Table 8 shows average results of all subtests used in the study.

**Table 7:** Level of the individual cognitive functions—differences between patients with schizophrenia (*N* = 128) versus the control group (*N* = 68)

Scale	Schizophrenia (*N* = 128)		Controls (*N* = 68)		Test of difference Sign.	
	M	sd	M	sd	*Test U*	*p*
WSCT correct answers	26,61	11,318	35,76	9,244	2019,5	0,000
WSCT total errors	36,37	11,821	27,37	7,191	2028,5	0,000
WSCT perseverative answers	25,05	17,749	15,05	7,643	2942,5	0,001
WSCT perseverative errors	20,66	15,737	12,88	5,776	2970,5	0,001
WSCT conceptual level	16,39	13,872	28,14	10,099	1995,5	0,000
WSCT categories achieved	0,66	,864	1,80	2,623	2091,5	0,000
WSCT to complete 1^st^ category	41,90	26,041	23,28	19,002	2754,0	0,000
WSCT failure to maintain set	0,66	1,022	0,94	0,864	3194,5	0,004
RAVLT A1-A5	26,84	11,124	43,51	10,437	1152	0,000
RAVLT A6	5,20	2,957	9,25	3,086	1509,5	0,000
RAVLT A7	4,25	2,694	8,81	3,021	1174,5	0,000
RAVLT list B	2,96	1,428	5,34	1,523	1104	0,000
RAVLT Recognition	,71733	,196041	,90170	,132401	1719	0,000
ROCFT similarity	,52	,501	,99	,122	2308,5	0,000
ROCFT main elements	3,89	2,040	6,36	1,366	1406,500	0,000
TMT A	81,01	58,753	32,84	14,624	1027	0,000
TMT B	274,73	298,46	67,74	31,338	886,5	0,000
Wechsler digits forward	6,11	2,185	7,84	1,920	2422,000	0,000
Wechsler digits backward	3,70	1,638	6,07	1,645	1360	0,000
Verbal fluency	10,17	4,864	16,72	4,836	1429,5	0,000
Stroop words	77,17	18,158	94,04	12,942	1921,500	0,000
Stroop colors	55,38	16,487	73,21	13,521	1651	0,000
Stroop Interferention	29,49	12,251	44,35	10,108	1458	0,000
Stroop Coefficient	31,7587	8,06665	40,9462	6,19602	1539	0,000

**Table 8:** Raw scores of individual subtests depending on age groups

	Age: 18-25		Age: 26–35		Age: 36–45		Age: 46–55	
	M (Schi)	M (Controls)	M (Schi)	M (Controls)	M (Schi)	M (Controls)	M (Schi)	M (Controls)
WCST total errors	33,09	28,59	33,50	28,55556	38,34	26,50	40,53	24,46
WCST pers. errors	22,21	16,36	24,81	16,94444	23,90	10,66	29,28	14,23
WCST categories ach.	0,96	2,27	0,87	1,38889	0,31	1,58	0,46	1,76
RAVLT A7	5,12	8,00	4,53	8,66667	4,28	9,0	3,06	10,25
RAVLT Recognition	0,77	0,86	0,73	0,92995	0,74	0,90	0,61	0,91
ROCFT main elements	4,25	6,04	4,37	6,80000	3,43	5,63	3,50	6,84
TMT A	72,28	30,50	62,78	33,57143	88,87	35,91	100,09	32,76
TMT B	247,53	61,45	175,21	76,57143	313,18	69,08	363,00	62,84
Digits forward	6,18	7,40	6,81	7,76190	5,75	8,36	5,68	8,23
Digits backwards	4,00	5,72	3,84	5,76190	3,68	6,36	3,28	6,92
Verbal fluency	9,37	15,54	10,90	17,15000	11,68	17,66	8,71	17,15
Stroop words	76,56	90,90	86,78	93,80	78,25	95,75	67,09	98,15
Stroop colors	54,84	71,63	62,71	71,66	54,62	75,08	49,31	76,61
Stroop Interferention	32,40	42,00	33,59	46,90	27,93	43,33	24,03	45,15
Stroop Coefficient	31,58	39,85	35,94	40,37	31,47	41,89	28,03	42,83

Significant differences were revealed between patients with schizophrenia and controls in all the domains of measured cognitive functions. The obtained results allowed for conducting further analyses, with the aim to compare the individual corresponding age groups to the clinical group and the control group.

### Comparison of corresponding age groups regarding cognitive functions

For further analyses subtests considered key in the individual tests were selected: the number of errors in WCST (attention and working memory impairments), number of perseverative errors in WCST (“on-line” processing impairment), number of completed sequences in WSCT (planning), RAVLT A7 delayed recall score (retrieval from memory), RAVLT recognition (ability to remember), main elements of ROCFT (visual memory), TMT B (planning and set-shifting), Wechsler's digits span forward (rote auditory memory), phonemic verbal fluency and Stroop's interference (inhibitory control) (see Tables 8 and 9).

**Table 9:** Significance of differences in individual age groups (schizophrenia and control group)—Mann–Whitney *U* test

Age		WCST total errors	WCST perseverative errors	WCST categories achieved	RAVLT A7	RAVLT Recognition	ROCFT main elements	TMT A	TMT B	Digits forward	Digit backwards	Verbal fluency	Stroop Coefficient
18–25	U	271,00	280,50	269,00	**179,50**	242,00	**162,00**	**121,50**	**86,00**	**235,50**	**185,00**	**111,00**	**149,00**
	*p*	0,157	0,210	0,147	**0,001**	0,053	**0,000**	**0.000**	**0.000**	**0,039**	**0,002**	**0,000**	**0,000**
26–35	U	**179,00**	224,00	195,50	**105,50**	**108,50**	**112,00**	**109,50**	**136,00**	255,50	**145,00**	**138,00**	**207,00**
	*p*	**0,028**	0,20	0,061	**0.000**	**0,000**	**0.000**	**0.000**	**0.000**	0.144	**0.000**	**0.000**	**0.018**
36–45	U	**65,00**	**82.50**	**31.00**	**48.00**	**73.00**	**75.00**	**41.00**	**34.00**	**58.50**	**33.00**	**64.50**	**372.00**
	*p*	**0.001**	**0.007**	**0.000**	**0.000**	**0.003**	**0.004**	**0.000**	**0.000**	**0.001**	**0.000**	**0.001**	**0.000**
46–55	U	**33.50**	**129.50**	**64.50**	**4.00**	**29.50**	**35.50**	**11.00**	**2.50**	**76.000**	**17.00**	**30.50**	**12.00**
	*p*	**0.000**	**0.048**	**0.000**	**0.000**	**0.000**	**0.000**	**0.000**	**0.000**	**0.000**	**0.000**	**0.000**	**0.000**

Bold print - Singificance *p* < 0.05

In Table 9 statistically significant differences were highlighted with bold print. The significant results in the Mann–Whitney U tests presented above can be read in two ways. Analyzing the data by columns will illustrate which age groups demonstrate significant differences with regard to specific cognitive functions Analyzing the data by rows will illustrate the number of differences within the various functions in an individual age group. Raw scores were presented in Table 8.

The obtained results seem to suggest that subjects with corresponding age groups differ significantly in level of cognitive functioning. Individuals with schizophrenia aged 26–35 years start to have noticeable difficulties in comparison to controls with correct performance in one subtest of WCST, while patients from subsequent age groups scored lower than controls in more subtests of WCST. Interestingly, there were no significant differences in WCST scores between patients and controls in the youngest groups, however, raw scores suggest that executive functions can be lower in this group of patients.

Significance of the differences between contiguous age groups of patients was analyzed. Patients aged 18–25 differed significantly from patients aged 26–35 only in number of words read in Stoop A subtest. Groups 26–35 and 36–45 differed from each other by score in Stroop test, TMT test, and number of retrieved elements in ROCFT (*p* < 0,05). RAVLT Recognition was the only subtest that differed the 36–45 and the 46–55 groups significantly (*p* = 0043). In every comparison scores of “older” groups were less favourable than “younger” groups.

In attempt to find potential sources of executive impairment, correlations of WCST and other variables were analyzed. Perseverative errors in WCST correlated significantly with PANSS Disturbance of volition (0.22), Sequences completed and conceptual level correlated with PANSS Preoccupation scale (0.18). Similar power of correlations was observed in relation of WCST Perseverative errors and number of months of 1^st^ untreated psychosis (0.20).

## Discussion

The neurocognitive tests results obtained in the present study confirm previous reports that schizophrenia is a disease in which generalized cognitive dysfunctions occur [35–37]. Cognitive disorders observed in patients with schizophrenia occurred in the majority of measured cognitive domains and the number of deficits were present across all age groups.

In patients with schizophrenia cognitive disorders (in comparison to their specific age group) were present in each of the basic cognitive domains such as verbal-linguistic learning, visual memory, inhibitory control, planning, set-shifting, rote memory, auditory memory, working memory and verbal fluency. Cognitive disorders appeared in patients in all age groups (18–55 years) and the patients performed the tasks less efficiently than healthy individuals. The obtained results confirm the results of other studies covering multiple domains of cognitive functioning [16, 37, 38].

The results of the present study may be seen as partly similar to other researchers’ conclusions. In several longitudinal studies [28] the progression of neuropsychological disorders associated with age was not confirmed. Present study suggests that patients with schizophrenia performed on the tasks significantly worse than healthy individuals, which suggests occurrence of cognitive deficits in early course of the disease, while raw scores indicate their relative stability across the age group. In terms of executive functions, other studies show their initial deficits before the disease onset, while they subsequently reach a stable level and later rapidly drop during in older age [39, 40]. The data collected for this article confirms that pattern. The youngest groups show slight, negligible differences, which become significant and stable as people in these groups age.

In one other research the deterioration of the results obtained in neurocognitive tests in patients aged 40–80 was observed mainly in the domains of verbal learning and processing speed [41]. In present study many more deficits were observed in every measured cognitive domains. The fact is consistent with other study that confirm faster cognitive deterioration in older patients with schizophrenia than in the control group [39, 40].

On the basis of the obtained data one may conclude that older patients with schizophrenia have relatively greater levels of cognitive impairment associated with age versus healthy individuals [42]. However, other authors claim that these effect is not connected with deterioration, but can rather be explained by poor engagement in care, access to treatment, treatment response or cumulative influence of substance abuse, deficits in cognition, and other psychosocial factors [43].

Collected data suggests that differences between clinical group and controls in executive functions of working memory and attention (errors in WCST) can be “more visible” in group of 26–35 years old, “later” than other cognitive disturbances that are present in all age groups, and therefore can be more “primary” than other. The next executive impairment (in comparison to controls) that is “more visible” in subsequent age group is “on-line” processing (perseverative errors) and planning (completed sequences).

Present study is also partially consistent with the study based on MATRICS consensus [44], in which patients with schizophrenia exhibited greater cognitive impairment than controls. In a cross-sectional study the authors revealed that patients early in the course of schizophrenia exhibited similar level of impairment compared to patients with a chronic schizophrenia. The data presented here focused on differences between patients and controls, shows that number of significant differences between inpatients and controls increase in every single “older” subgroup (similar age). At the same time, most cognitive functions show a slight connection to the age and the time of disease onset whereas perseverative errors (cognitive rigidity) seem to be associated with the difference between the time when the disease begun and the number of years since the first hospitalization. The longer the period of time spent without hospitalization the greater the cognitive rigidity shown in the study. It may be connected with fixed deficits caused by a long period when patients were not treated, at least in some cases.

One significant drawback of the present study is the fact that it was cross-sectional. Thus, it is only possible to analyze the differences between healthy individuals versus patients with schizophrenia in the individual age groups. Other shortcoming is linked to the fact that only inpatients were examined, the outcomes are thus limited, because of little likelihood of qualification to research in recovered patients.

## Conclusions

The conclusions of the present research indicate the possibility of the occurrence of primary impairments of verbal-learning, memory, shifting, inhibitory control and verbal fluency, which persist in older age groups, and it is possible that executive impairment are “less visible” in early course of disease. Further longitudinal study should confirm if executive functions lower significantly in group of patients aged 36-45.

## Abbreviations

MADRS: Montgomery-Asberg Depression Rating Scale
PANSS: Positive and Negative Syndrome Scale
RAVLT: Rey Auditory Verbal Learning Test
ROCFT: Rey-Osterrieth Complex Figure Test
TMT: Trail Making Test
WCST: Wisconsin Card Sorting Test
